# Supplementary material for: Zygote structure enables pluripotent shape-transforming deployable structure
Source: PNAS Nexus. 2023 Mar 14;2(3):pgad022. doi: 10.1093/pnasnexus/pgad022 (PMC10013337; doi:10.1093/pnasnexus/pgad022)
Supplement: pgad022_Supplementary_Data [file pgad022_supplementary_data.zip › PNASNEXUS-PNASNEXUS-2022-00977-T-s10.pdf]

Supporting Information for
Zygote structure enables pluripotent shape-transforming deployable structure

Yu-Ki Lee[1†], Yue Hao[2†], Zhonghua Xi[2], Woongbae Kim[3,4], Youngmin Park[1], Kyu-Jin Cho[3,4], Jyh-Ming Lien[2*], In-Suk Choi[1*]

[1]Department of Materials Science and Engineering, Research Institute of Advanced Materials (RIAM), Seoul National University, Seoul, 08826, Republic of Korea

[2]Department of Computer Science, George Mason University, Fairfax, VA, 22030, USA.

[3]Soft Robotics Research Center, Seoul National University, Seoul 08826, Republic of Korea.

[4]Department of Mechanical Engineering, Institute of Advanced Machines and Design, Institute of Engineering, Seoul National University, Seoul 08826, Republic of Korea.

*** Corresponding Authors:** Jyh-Ming Lien and In-Suk Choi.

Email: jmlien@cs.gmu.edu and insukchoi@snu.ac.kr

This PDF file includes:

Figures S1 to S13
Legends for Movies S1 to S7
SI References

Other supporting materials for this manuscript include the following:

Movies S1 to S7

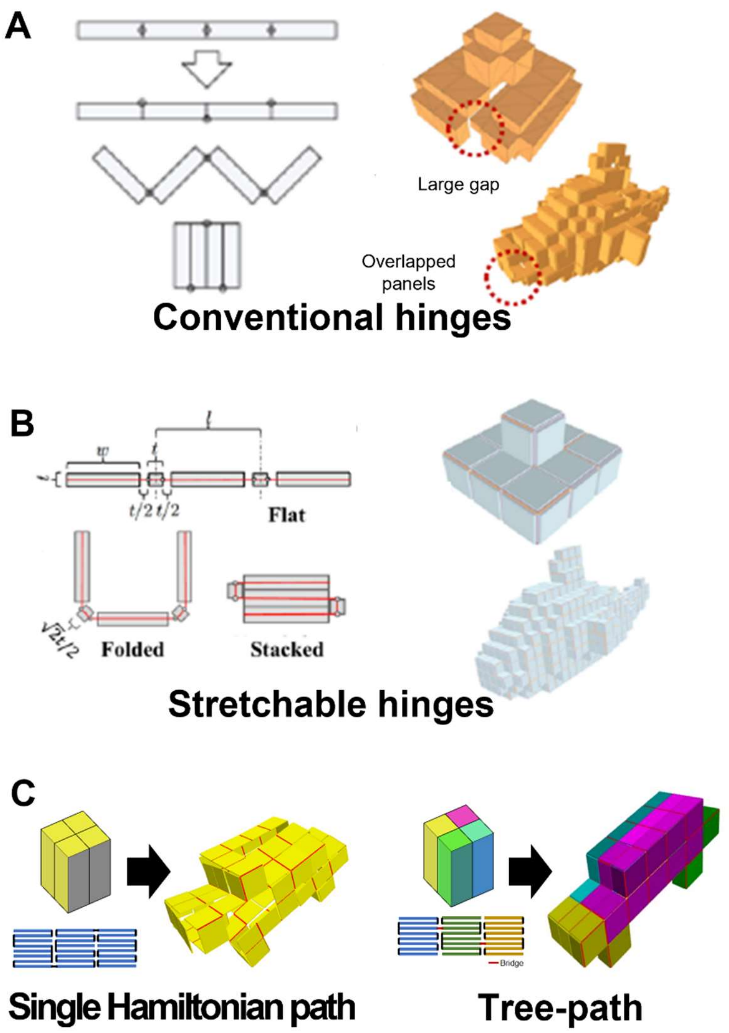

Fig. S1. Folding error caused by thick *origami*. (A) There were lots of studies about designing the rotational hinges that can fold thick panels in *origami* tessellation design called thick *origami*. Given a flat structure tessellated by thick panels, the most common approach is axis-shift method. (1) This method shifts each rotation axis to either top or bottom of the thick panel depending on the crease type, i.e., mountain or valley. However, applying the axis-shift hinges in our pluripotent evolving structure results in gaps and self-intersections since our system requires a full range of folding angle from $-\pi$ to π. **(B)** In our algorithm, we used a new thickness accommodation method based on Offset Crease (2) in our algorithm. We take the folded state as the initial state, make the center of the thick panels overlap with that of zero-thickness material so the panels can be folded in both directions. For a given $(l-2t) \times (l-2t) \times t$ size panel where l is the length and t is the thickness of panel, the length of our linear hinge h should be

$$h = \begin{cases} cos\left(\frac{\theta}{2}\right) \cdot t, & |\theta| \leq \frac{\pi}{2} \\ \frac{\sqrt{2}}{2} \cdot sin\left(\frac{|\theta|-\frac{\pi}{2}}{2}\right) \cdot t, & |\theta| > \frac{\pi}{2} \end{cases}$$

where θ is the folding angle of an ideal crease, and t is the thickness of the panel. When $\theta = 0$, the flat state, the hinge length $h = t$, and when $\theta = \pm\pi/2$, in the maximum folded state, the hinge length $h = \sqrt{2}t/2$. Finally, when $\theta = \pm\pi$, the stacked state, the hinge length $h_i = t$. During the

entire folding range, we have $\sqrt{2}t/2 \leq h_i \leq t$. **(C)** However, realizing this stretchable and robust hinge is challenging. For hinges with small Gaussian errors (0.01 rad standard deviation), a coded sequence for single Hamiltonian path causes large inaccuracy for deployed 3-D car model with 92 panels. Our practically solve this problem by introducing tree-stacking algorithm that connect panels in tree-path rather than single stripified path (i.e., single-chain structure). A coded sequence for tree-path reduces cumulative errors by shortening the average length of chain components.

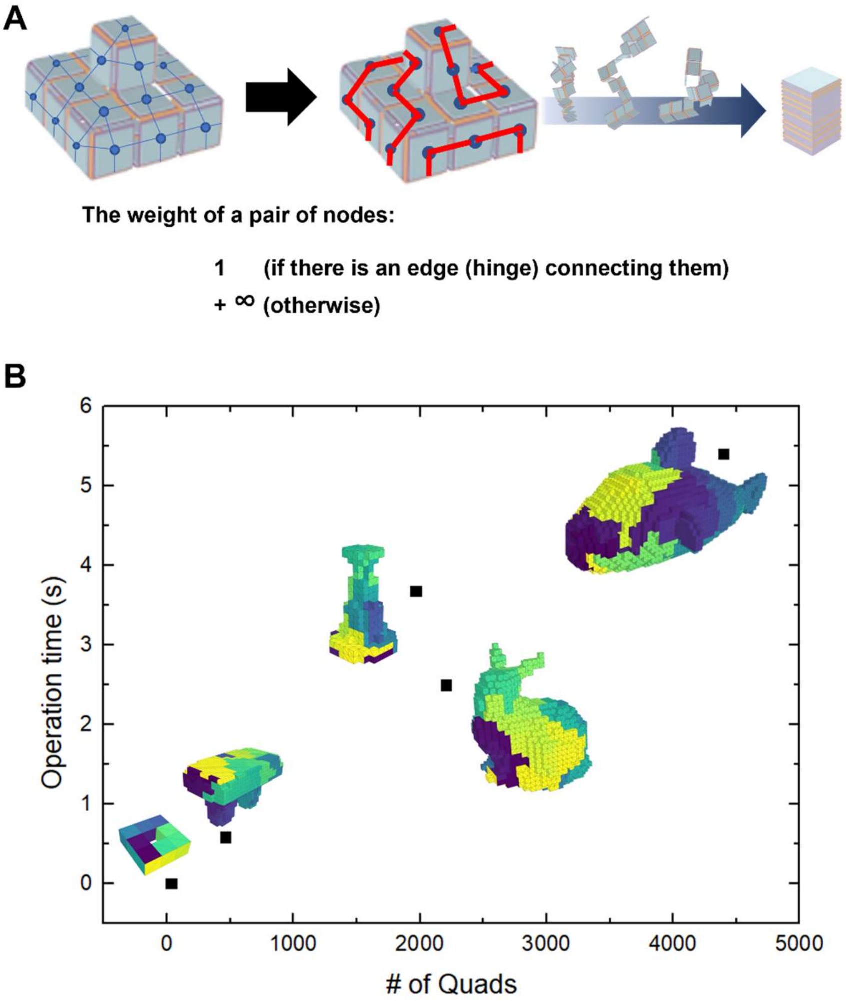

Fig. S2. Process of finding the single Hamiltonian path by TSP algorithm. (A) In our stacking system, the quads are regarded as vertices in a graph and are only connected with four adjacent quads by the edges having the weight as 1. Fortunately, if the graph represents a quad mesh, such as our panel network, it is known that a Hamiltonian cycle of its triangulation can be computed in linear time. Although no existing methods are suitable for creating stackable strip, it is known that Hamiltonian cycles must exist in the dual graph of the voxelized mesh as a 4-regular graph such that we can cut the mesh and make it stackable. (3) We convert the Hamiltonian path problem (HPP) into the well-known Traveling Salesman Problem (TSP), which finds a Hamiltonian cycle with minimum cost (the sum of crossed dual edge weights). (4) There are several reasons for solving TSP instead of HPP: 1) TSP is a special case of HPP, and a solution to TSP is always a solution to HPP. 2) TSP is a well-studied problem comparing to HPP. Therefore, many efficient solvers are available in the public domain. 3) The known upper bound n helps the solver to cut unnecessary branches, so the solver could find solutions more efficiently. We set the weight of a pair of nodes to 1 if there is an edge (hinge) connecting them and +∞ otherwise. Then, we use a state-of-art traveling salesman problem (TSP) solver named Concorde TSP solver that can find Hamiltonian cycles efficiently with time growing almost linearly to the number of quads in the mesh. **(B)** We verified that the running times grow almost linearly to the number of quads *N* which means that our concept is practically applicable to even complex 3-D models approximated with thousands of panels.

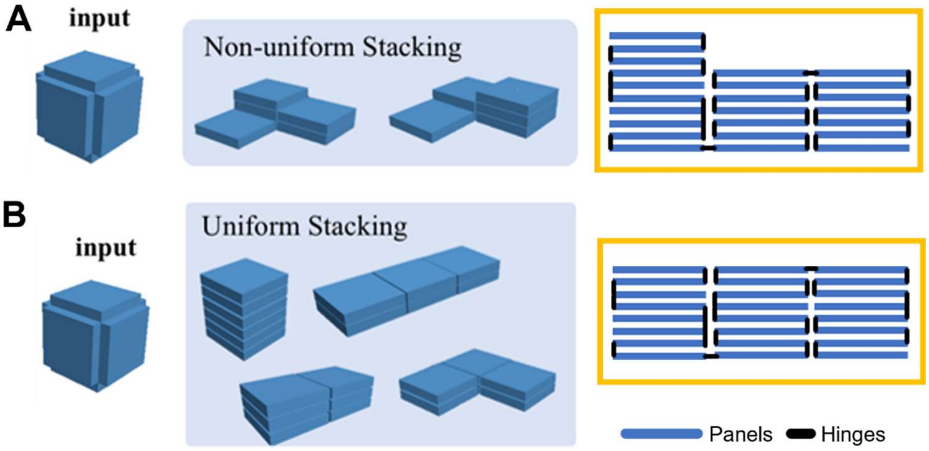

Fig. S3. Compaction of stripified panels. (A) Stripified panels can be more compacted with multiple piles, however, it is not always possible that all the piles have the same number of panels, which we call uniform stacking. In our compaction process, we say a pile is an uphill pile if the heights of its panels along the strip are increasing, otherwise, it is a downhill pile. The base panel of a pile is the panel with the height of 0. A panel is the roof panel of the pile if it is the highest one in that pile. Assuming we always start stacking with an uphill pile then an exception can be made for the last pile if it is an uphill pile. The last uphill pile can have different height, either higher or lower than the rest of the piles. For valid stacking (i.e., base panels of all piles touch the ground), each pair of uphill and downhill piles need to have the same height, while the downhill to uphill pair of piles can have different heights. For simplicity, the height of the later uphill pile is chosen from $\{h, h \pm l\}$, where l can be $1, 2, \cdots, m, m < h$. This gives us $m \cdot \left(3^{\lfloor k/2 \rfloor} - 1\right)$ different stacked states for each strip. However, this constraint can generate non-uniform stacking. **(B)** For uniform stacking, given *N* panels and the number of piles *K*, the height *h* of each pile should be $\lceil N / K \rceil$. By breaking the Hamiltonian cycle at different locations (Note that we find a Hamiltonian cycle and break it, rather than find a Hamiltonian path), we have up to *N-1* different strips, and each strip defines one single uniform stacking. However, not all stacked states are feasible since some piles might collide with others. We refer to these self-intersecting states as infeasible state and the feasibility can be validated in time linear to the size of the panels. Non-uniform stacking can also be used for our pluripotent evolving structure as demonstrated in Fig. S8.

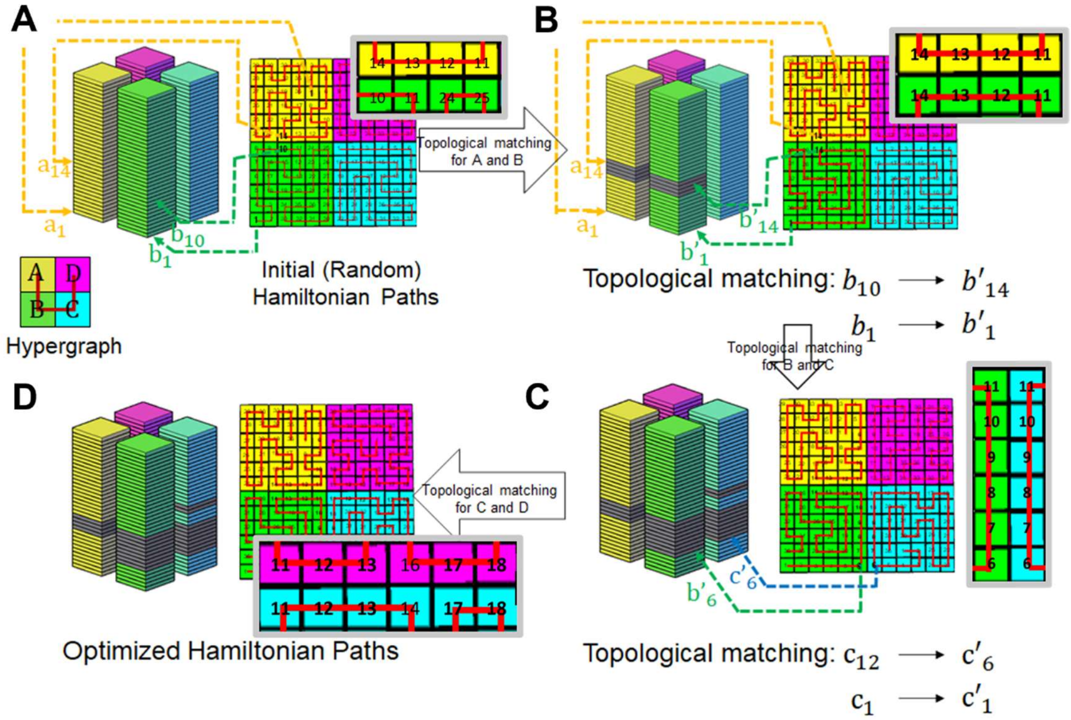

Fig. S4. Topological matching process. (A) Depending on topological distance from $v_{hype\ ,r}$ (colored yellow) in G_{hyper}, we recursively match the two neighboring Hamiltonian paths. For example, in our plane sheet having 144 panels case, we match the Hamiltonian paths of the yellow and green partitions first, then those of the green and sky-blue partitions and then those of the sky-blue and purple partitions are matched. **(B)** To match the Hamiltonian paths on the yellow and green partitions, we first fix the Hamiltonian path on the yellow partition. Then, we set a new Hamiltonian path on the green partition under the condition that there should be at least one panel that is adjacent to a panel in the yellow partition and has the same floor. **(C)** The twelfth panel in the sky-blue partition is changed with the sixth panel and that a new Hamiltonian path is generated in this partition. **(D)** By repeating the same process on the remaining partitions, topology of all the Hamiltonian paths is adjusted to be connectable in both stacked and deployed states. (5)

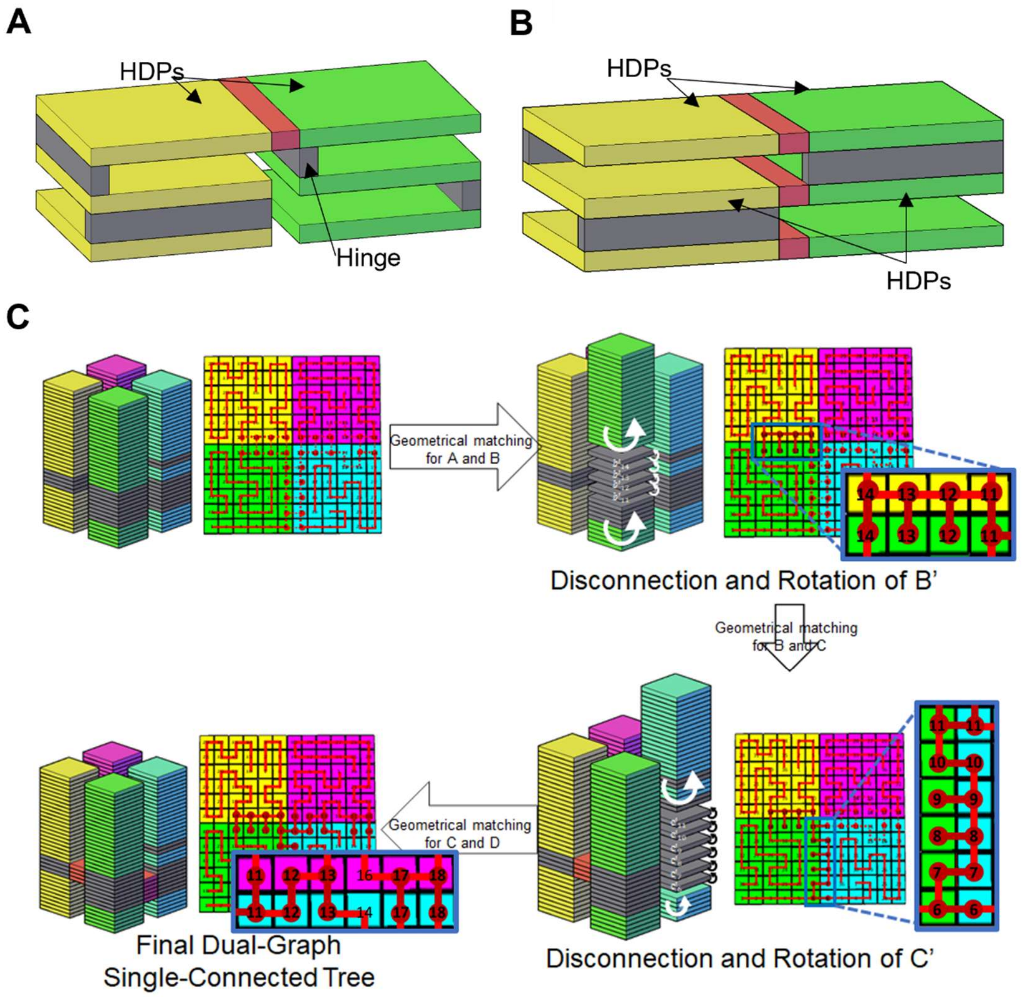

Fig. S5. Geometrical matching process. (A) Topological matching process can potentially generate nonmanifold geometries or **(B)** self-locked configuration that stacked panels cannot deploy into a 3-D structure. **(C)** Our geometrical matching process check these potential problems and fix it by breaking the path and changing the orientation of such cut panels while all panels become deployable. Like the topological matching process, this process also recursively fixes the potential problem between neighboring piles according to the hypergraph. (5)

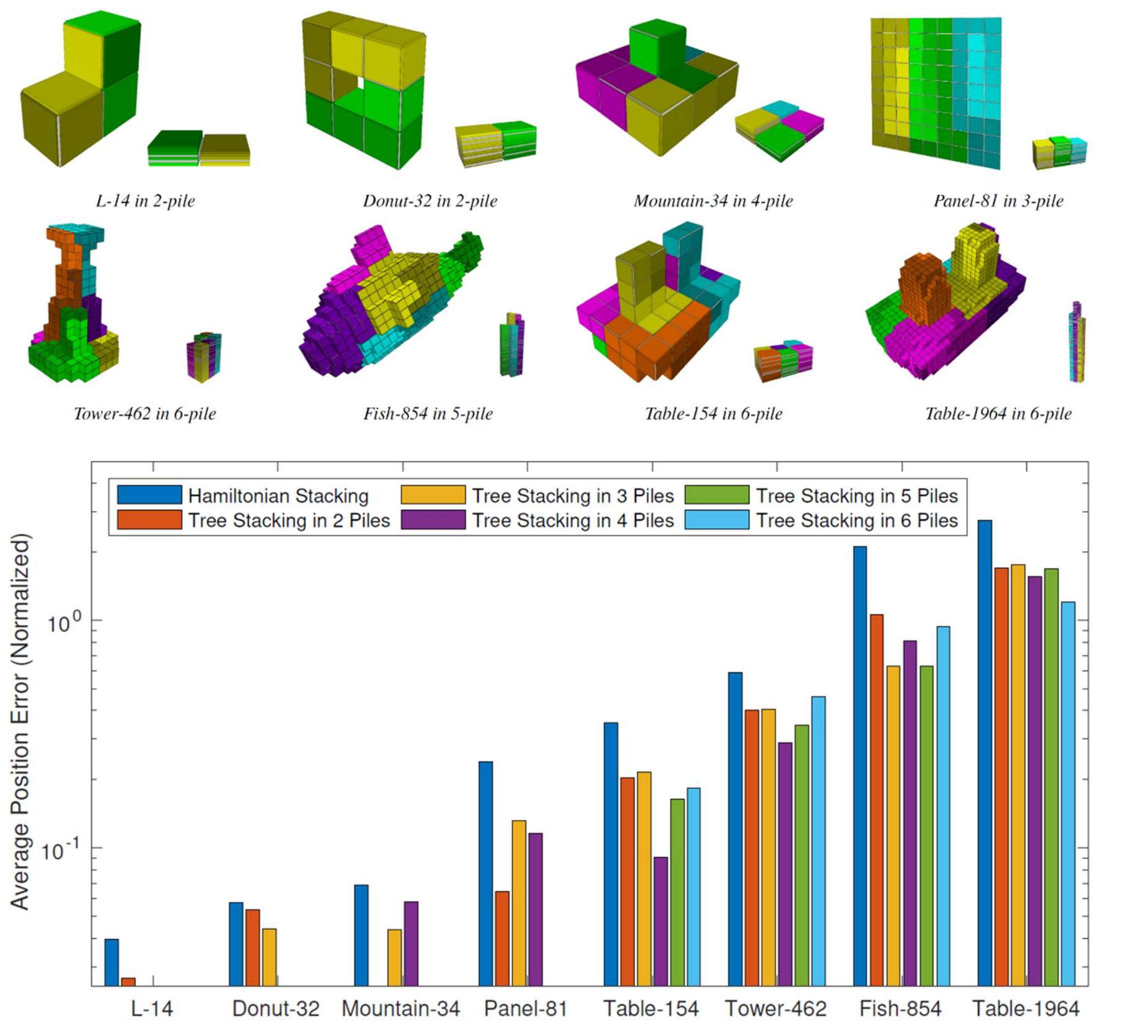

Fig. S6. The folding position errors simulated by adding small Gaussian noise (zero mean and 0.01 rad standard deviation) to the hinge angles, averaged over the number of quad panels in the model. The topology of the zygote structure is closely related to the folding accuracy, as a long chain structure suffers cumulative control errors in the forward kinematics. In our previous study on maximizing the number of HDNs, which exponentially reduces the length of the chain components in the structure rather than guaranteeing equal partitioning, we verified that the folding errors were exponentially reduced with the tree-stacking approach compared with the naïve algorithm finding a single Hamiltonian path. We added Gaussian noise (zero mean and 0.01 rad standard deviation) to the target hinge angles in the simulation. Hamiltonian stacking with a single long chain component results in a normalized length of 1.0, while tree stacking leads to consistently shorter chain components regardless of the geometry of the model. (5) Intuitively, the position error (folding error after deployment) increases if the distribution of the length (i.e., the number of panels) of each pile is large. This implies that the zygote structure, in which all piles have the same length, has the minimum average position error (folding error). Reprinted by permission from John Wiley & Sons Ltd. From Yue Hao et al., “Compacting Voxelized Polyhedra via Tree Stacking”, Computer Graphics Forum 38 (2019).

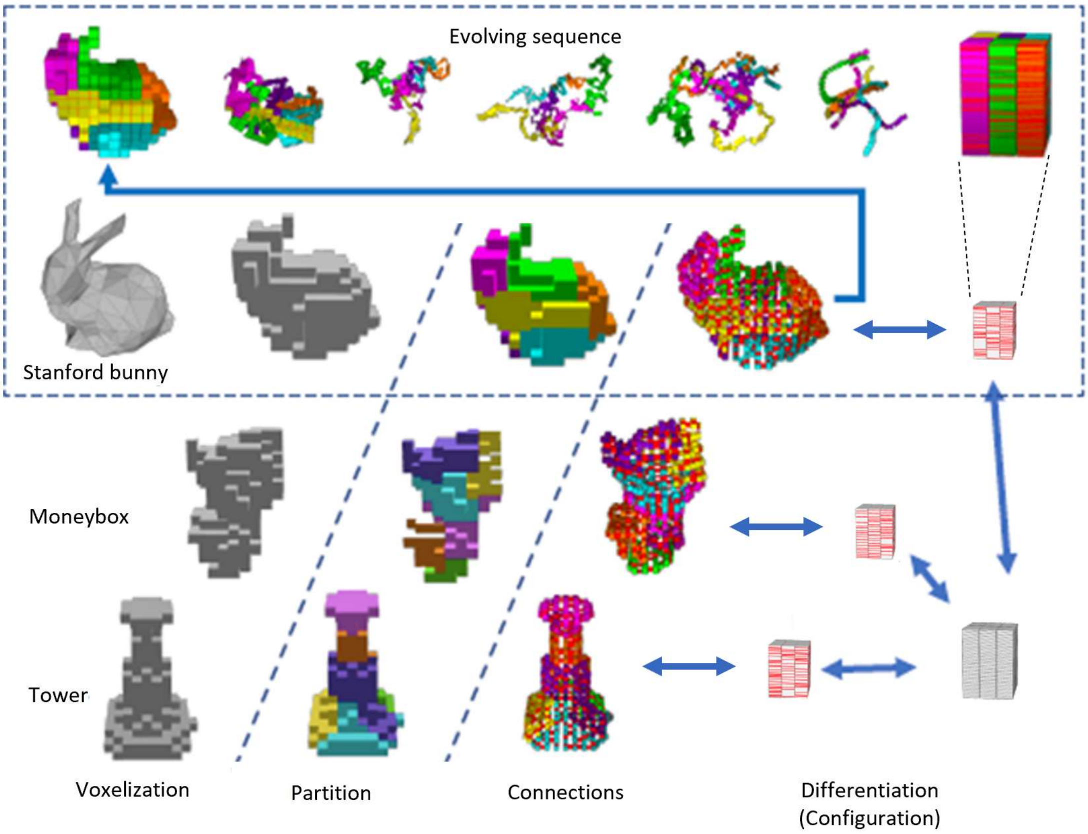

Fig. S7. Results for 498 panels. A zygote structure having 498 panels can deploy into a bunny, a moneybox, and a tower model. Our tree-stacking algorithm were valid in broad range of resolution from less than a hundred (Fig. 3 and Fig. 4 in main text) to thousands of panels (Fig. 2 in main text).

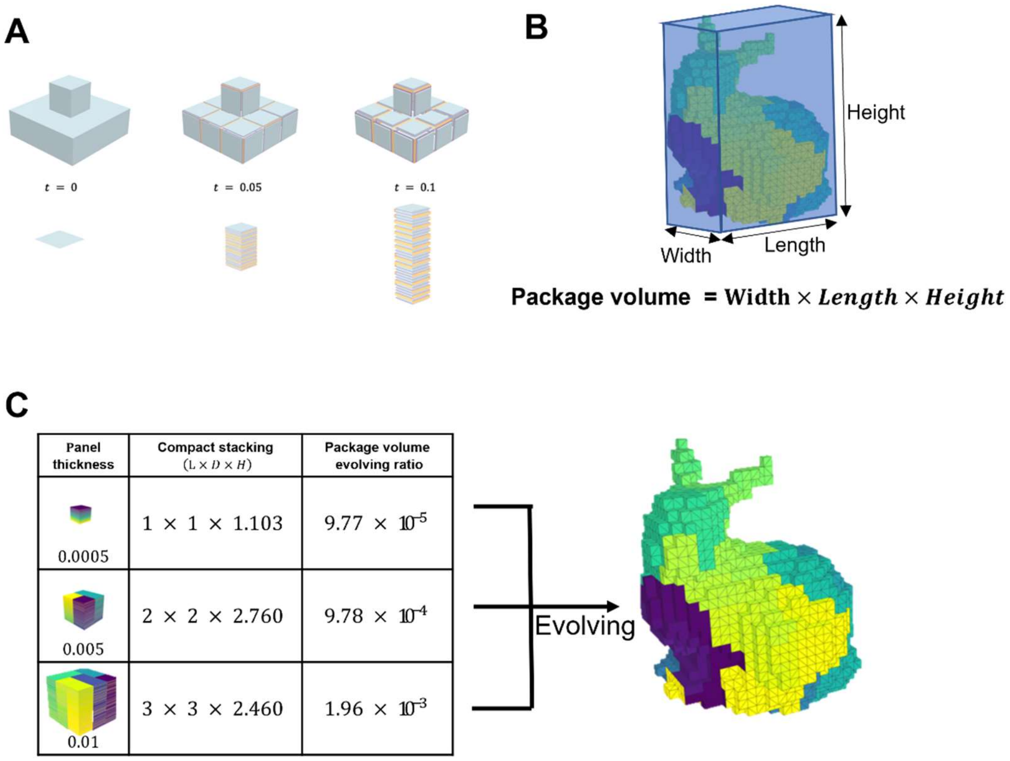

Fig. S8. Definition of the package volume expansion (VER). For panels of the same thickness and size, a zygote structure with many panels can be transformed into larger structures, e.g., 6 panels can form a cube (1x1x1 size), and 10 panels can form a parallelepiped shape (2x1x1 size). The scale expandability is determined based on the number of panels in the zygote structure, the thickness of each panel, and the geometry of the target 3-D shape. **(A)** For the same number of panels and the same target 3-D geometry, the thickness of the panel can determine the volume compaction/expansion ratio between the folded and stacked states. To quantitatively compare the volume compaction/expansion ratio, we used package volume that is widely used practically in shipping industry. **(B)** Package volume measure the practical size for packing an object in a cuboid box and. Package volume expansion ratio (VER) means the ratio of volume of the bounding box (Length × Width × Height) between the stacked and evolved states. **(C)** The thickness of panel can significantly affect to the VER.

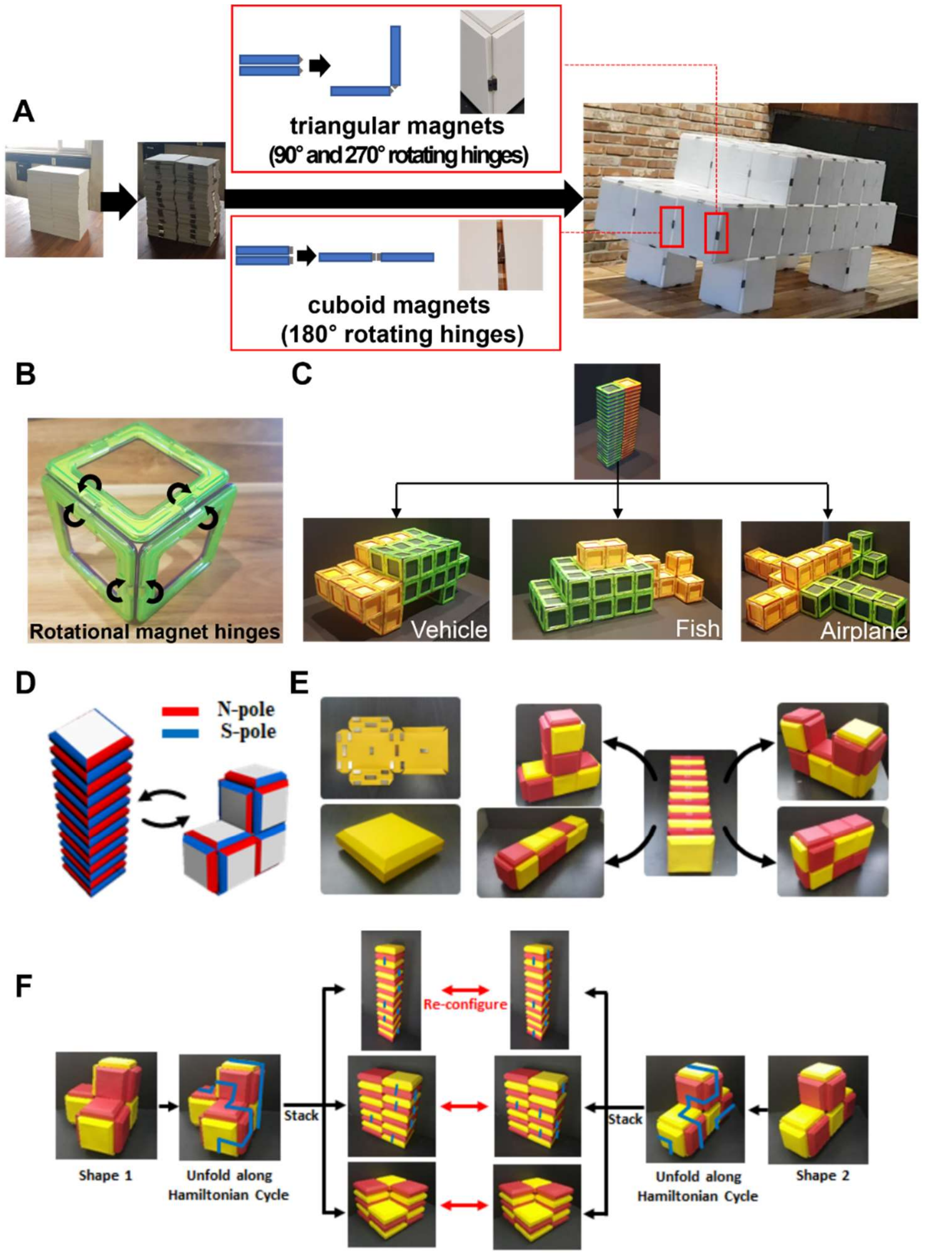

Fig. S9. **Experimental demonstration with magnetic hinges. (A)** We simply demonstrated our concept by attaching triangular (right angled triangle that the longest length is 1cm and the length of the triangular prism is 3 cm) magnets or cuboid magnets (3 cm × 1 cm × 0.5 cm) on sides of panels (20 cm × 20 cm × 1 cm size) as shown in Fig. 3C. **(B)** In addition, we also demonstrated it more easily with commercial toy panels embedding rotational magnets. **(C)** These panels enable reconfiguration of the zygote structure without additional process for re-arranging the hinges (i.e., detaching and re-attaching the hinges). **(D)** Furthermore, we verified that magnetic panels that each side has different pole can compactly stacked and evolves. **(E)** We fabricate a unit block using papers and commercial magnets and could transform the zygote structure consisting of these magnetic panels into diverse structures. Since all of side faces attracts each other, the final structures are robust and did not collapse. **(F)** Not only uniform stacking (Fig. S3), but non-uniform stacking can also be used for our pluripotent evolving structure as two different 3-D structures can be stacked into both uniformly stacked and non-uniform stacked states.

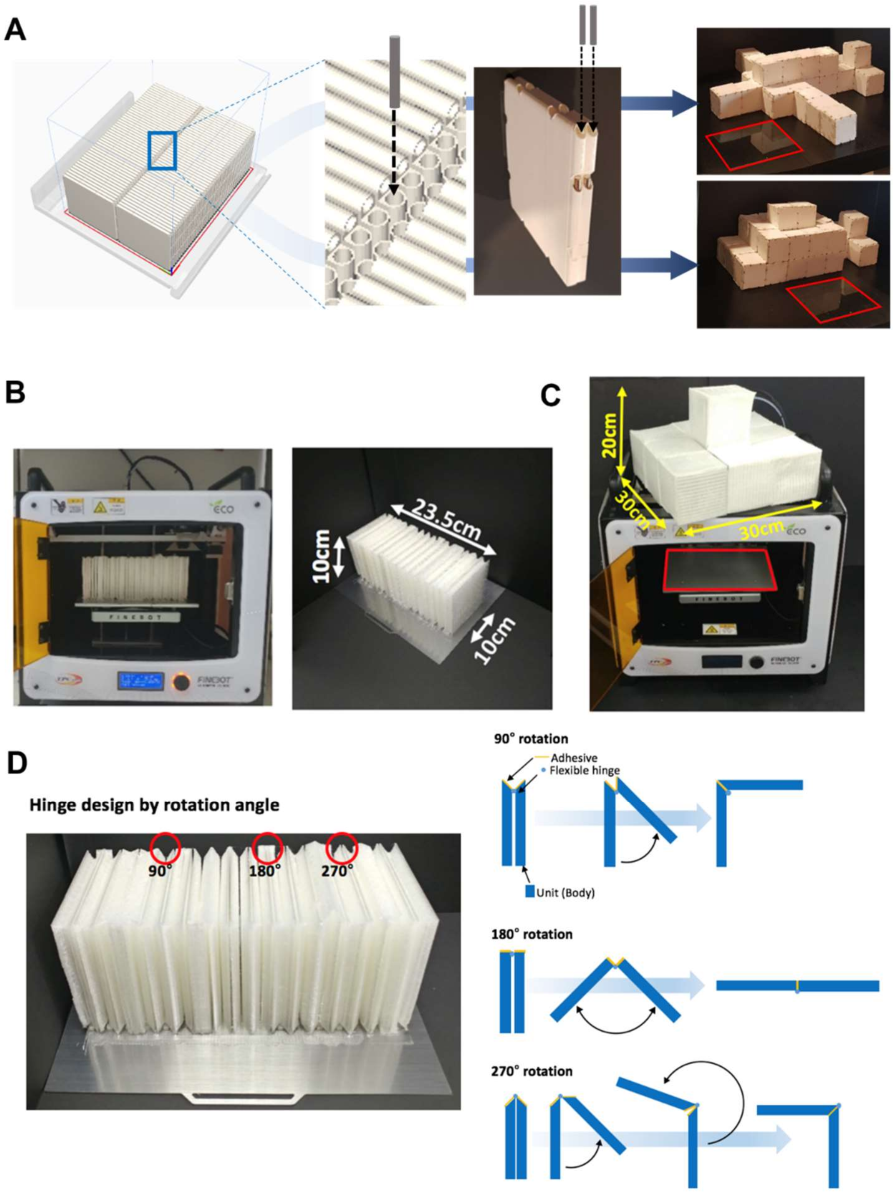

Fig. S10. Panel and hinge design for 3-D printed pluripotent evolving structure. (A) With our panel design, it is possible to insert cylindrical magnets even inside side faces of the printed panels, which enables reconfiguration of the zygote structure without additional process for re-arranging the hinges (i.e., detaching and re-attaching the hinges). **(B)** Otherwise, it is also possible to print the zygote structure that their connection path is already determined. For example, we could print panels and hinges at the same time and **(C)** deploy it without additional hinges-attaching process (we simply put glues on hinges for attachment between panels). **(D)** For this process, we designed two kinds of triangular hinges for 90° or 270° rotation and cuboid hinges for 180° rotation that are attached on panels before printing. With this simple and rational design of printed hinges, all panels and three types of hinges can be composed of single printed material (PLA). The flexibility of hinge can simply be controlled by the thickness control of the printed PLA material (flexible hinge part colored in sky-blue).

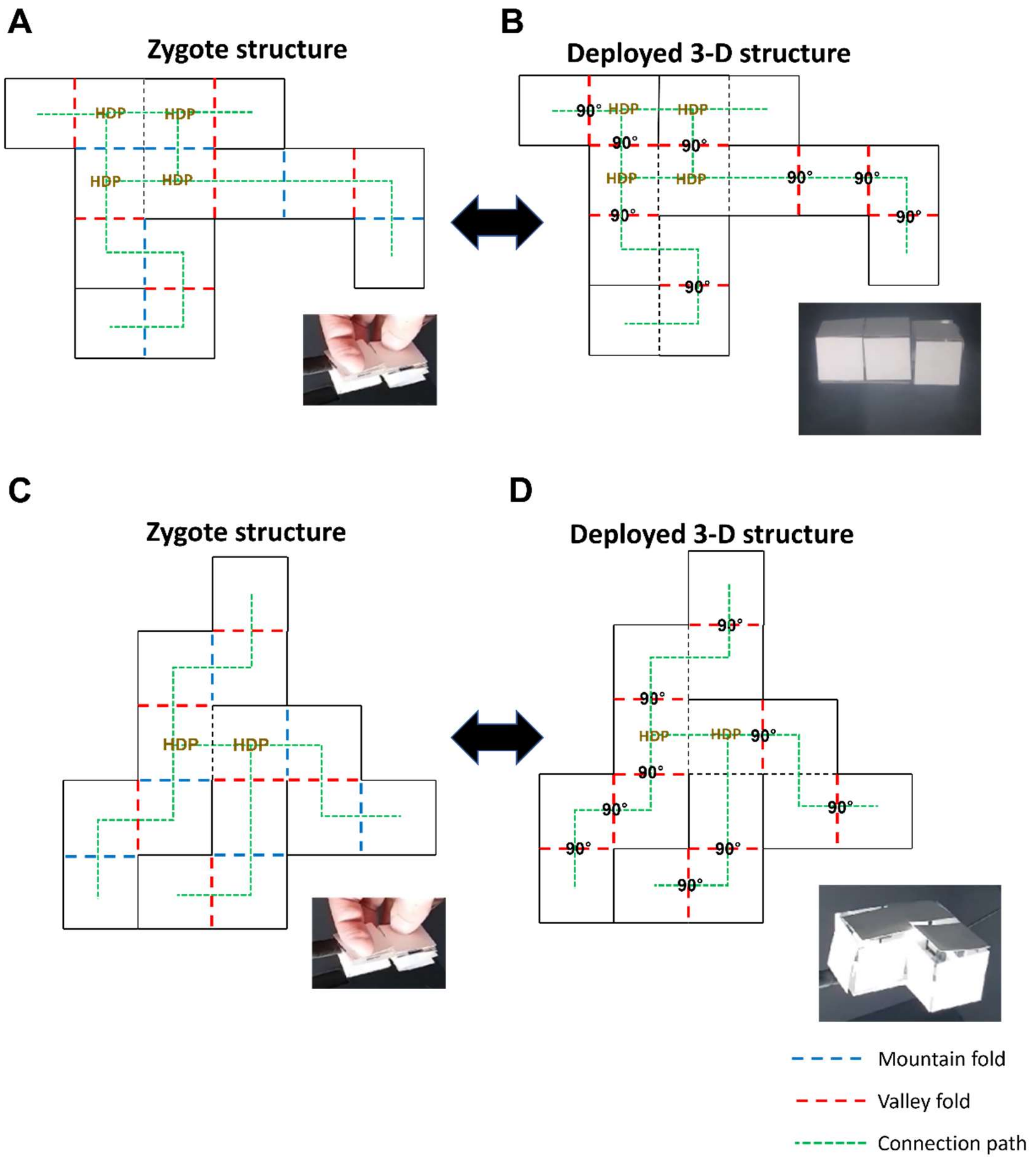

Fig. S11. Flattened planar figure of the zygote structure. (A) For a given a planar figure corresponding to a valid connection path for an I-shaped 3-D structure (shown in Fig. 4I), stacking all the panels by repeating mountain and valley folds starting from a root panel can develop a zygote structure. **(B)** The same planar figure can be transformed into the target 3-D shape with valid fold lines. The numbers on each crease line denote the angles required to transform the planar figure into the target 3-D shape. In the same way, a planar figure with the connection path for an L-shaped 3-D structure can be transformed into **(C)** the zygote structure and **(D)** the target 3-D shape based on the valid fold lines. Note that flattening the 3-D structure onto a 2-D plane along the connection paths may result in overlapped panels, as described in Fig. 2K and Fig. 2L.

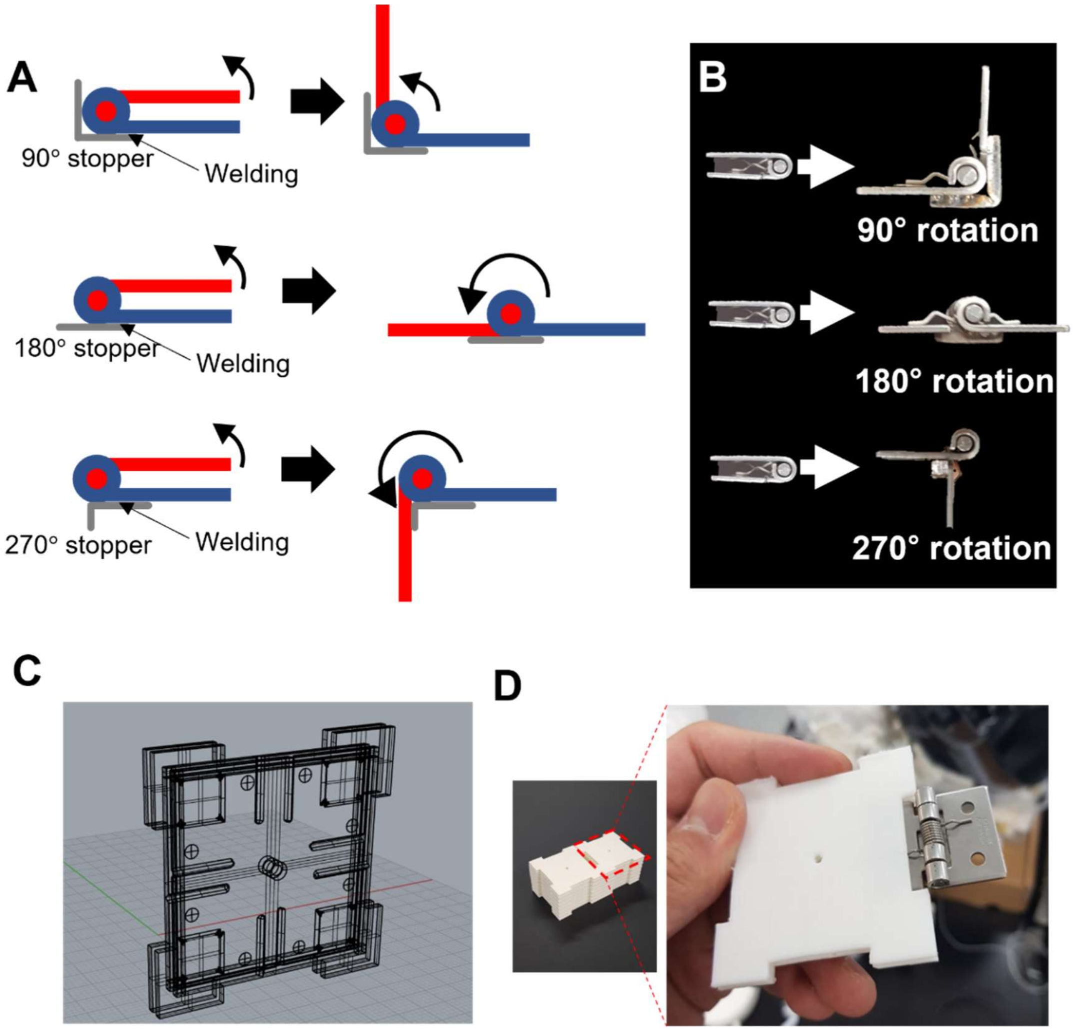

Fig. S12. Design of hinge and panels for experimental demonstration using commercial spring hinges. (A) We designed stoppers that can restrict the rotating angle of the spiring hinge at 90°, 180°, or 270°, and **(B)** fabricated them by welding stainless steels bars on the springs. **(C)** We also designed a panel that a commercial rotating hinge can be inserted to the side faces, and **(D)** fabricated them with a commercial 3-D printer.

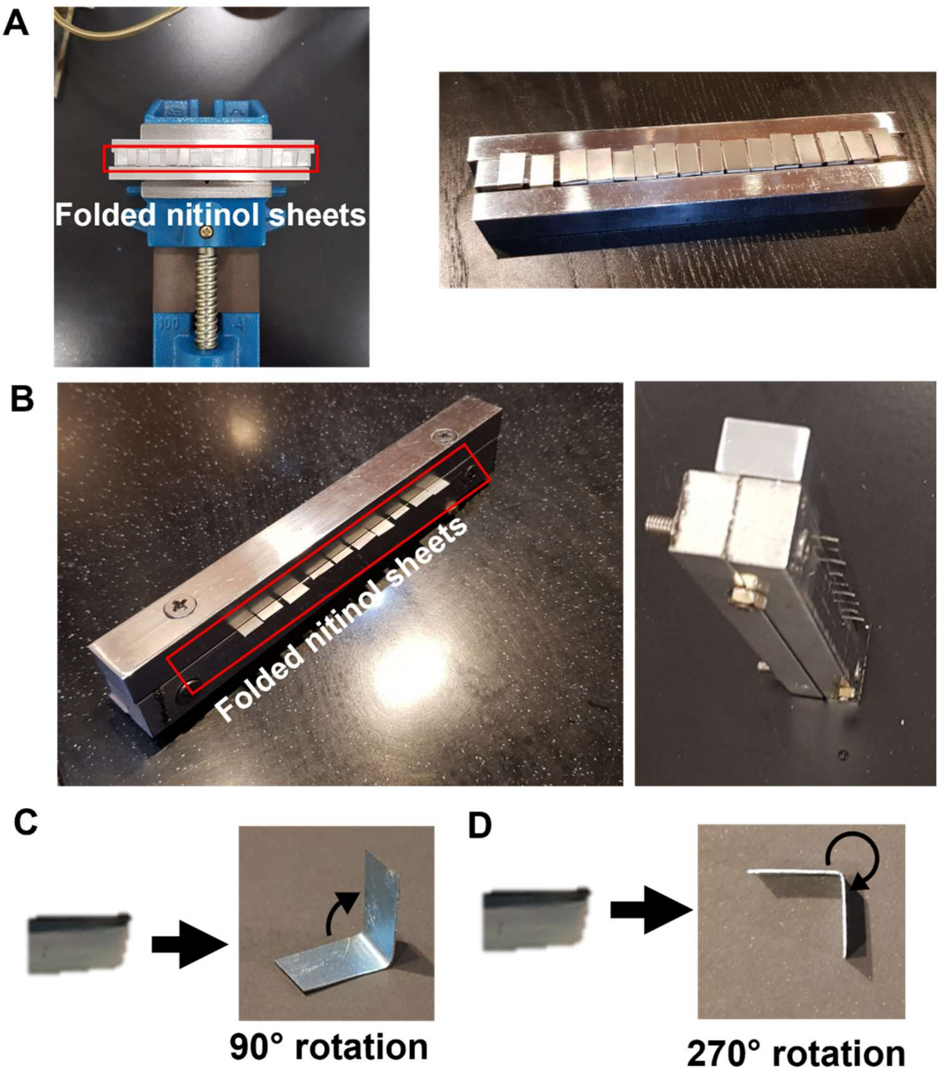

Fig. S13. Process of preparing shape memory alloy (SMA) hinges. (A) We used 0.125mm thick nitinol Foil (bought from AVENTION Co.,Ltd.). We fixed them with steel bars and folded the SMAs in 90° using a vice. **(B)** Then, we cover them with additional steel bar to prevent distortion during the annealing process. Annealed in 450 °C for an hour and quenched in water, we could program the shape of SMA. By folding the programmed SMA sheets with **(C)** 90° or **(D)** 270° (overlapping the two faces in both shapes), we could prepare the rotational hinges.

Movie S1 (separate file). Stacking diverse structures with a single Hamiltonian path and its potential application for a shape-reconfigurable structure.

Movie S2 (separate file). Stacking a plane sheet and a fish model (total 144 panels, respectively) into the same zygote structure with the tree-stacking algorithm.

Movie S3 (separate file). Zygote structure consisting of 4,000 panels that evolves into a chicken, a vehicle, and a fish, respectively.

Movie S4 (separate file). Zygote structure consisting of 92 panels that evolves into a vehicle, a fish, and an airplane, respectively.

Movie S5 (separate file). Demonstration of the zygote structure with plastic panels and magnets.

Movie S6 (separate file). Demonstration of the self-deployable zygote structure with energy releasing commercial spring hinges.

Movie S7 (separate file). Demonstration of the self-deployable zygote structure with shape memory alloy (SMA) hinges.

SI References

1. T. Tachi, Rigid-foldable Thick *Origami*, *Origami 5* (CRC Press, 2016).
2. S. A. Zirbel, R. J. Lang, M. W. Thomson, D. A. Sigel, P. E. Walkemeyer, B. P. Trease, S. P. Magleby, L. L. Howell, Accommodating thickness in *origami*-based deployable arrays, *J. Mech. Des.* 35 (2013).
3. E. Lemus, E. Bribiesca, E. Garduño, Representation of enclosing surfaces from simple voxelized objects by means of a chain code. *Pattern recognition* 47.4, 1721-1730 (2014).
4. D. Applegate, R. Bixby, V. Chvatal, W. Cook, Concorde TSP solver (2006).
5. Y. Hao, J.-M. Lien. Compacting voxelized polyhedra via tree stacking. *Computer Graphics Forum* 38 (2019).
